# Supplementary material for: Potential energy of complex networks: a quantum mechanical perspective
Source: Sci Rep. 2020 Oct 27;10:18387. doi: 10.1038/s41598-020-75147-w (PMC7592062; doi:10.1038/s41598-020-75147-w)
Supplement: Supplementary file 1 — Supplementary Information 1. [file 41598_2020_75147_MOESM1_ESM.pdf]

Supplementary Information

Potential energy of complex networks: a quantum mechanical perspective

Nicola Amoroso[1,2,+], Loredana Bellantuono[3,+], Saverio Pascazio[3,2,*], Angela Lombardi[2], Alfonso Monaco[2], Sabina Tangaro[2], and Roberto Bellotti[3,2]

[1]Dipartimento di Farmacia-Scienze del Farmaco, Università degli studi di Bari "A. Moro", I-70125 Bari, Italy
[2]Istituto Nazionale di Fisica Nucleare, Sezione di Bari, I-70126 Bari, Italy
[3]Dipartimento Interateneo di Fisica "M. Merlin", Università degli studi di Bari "A. Moro", I-70126 Bari, Italy
[*]saverio.pascazio@ba.infn.it
[+]these authors contributed equally to this work

Dressing transformations to reconstruct potentials from spectra

We rapidly sketch the method we adopted for reconstructing a potential in a 1D Schrödinger equation and the corresponding eigenfunctions from a set of given energy levels. Let us consider a one-dimensional symmetric potential $V(x)$, defining the Hamiltonian $H = p^2 + V$, with $p = -\mathrm{i}\partial_x$ (and $\hbar^2/2m = 1$). Suppose that E is the ground state energy of H. Let us now consider an arbitrary $\bar{E} < E$: since $\bar{E}$ cannot be an eigenvalue, the equation

$$(p^2 + V(x))F(x) = \bar{E}F(x) \tag{S1}$$

can be solved only by non-normalizable functions. In particular, the equation admits a symmetric solution ($F'(0) = 0$) with no nodes, whose inverse logarithmic derivative $f(x) = -F'(x)/F(x)$ satisfies the nonlinear equation

$$f'(x) - f^2(x) + V(x) = \bar{E}, \quad \text{with } f(0) = 0. \tag{S2}$$

It is now possible to associate to V a new potential $\bar{V}$, defined by

$$\bar{V}(x) = f'(x) + f^2(x) + \bar{E}, \tag{S3}$$

and check that the function

$$\Psi(x) = \Psi(0)\exp\left(\int_0^x f(y)\mathrm{d}y\right) \tag{S4}$$

is an eigenfunction of $\bar{H} = p^2 + \bar{V}$ with eigenvalue $\bar{E}$. Moreover, being the inverse of the solution $F(x)$ of Eq. (S1), it is symmetric and has no node, hence corresponding to the ground state of $\bar{H}$.

The above properties can be iteratively used to build a potential $V_1(x)$ characterized by the given set of discrete energy levels $\{E_n\}_{1\leq n\leq \widetilde{N}}$, with $E_n < E_{n+1}$, where E_1 is the ground state energy. The procedure can start from a constant potential $V_{\widetilde{N}+1} > E_{\widetilde{N}}$, so that the largest energy $E_{\widetilde{N}}$ falls below the (continuous) spectrum of $p^2 + V_{\widetilde{N}+1}$. At each step, one determines the solution f_n of

$$\begin{cases} f_n'(x) - f_n^2(x) + V_{n+1}(x) - E_n = 0 \\ f_n(0) = 0 \end{cases} \tag{S5}$$

and then updates the potential as

$$V_n(x) = f_n'(x) + f_n^2(x) + E_n = V_{n+1}(x) + 2f_n'(x). \tag{S6}$$

It is also possible to verify that

$$\psi_1^{(n)}(x) = \psi_1^{(n)}(0)\exp\left(\int_0^x f_n(y)\mathrm{d}y\right) \quad \Rightarrow \quad (p^2 + V_n(x))\psi_1^{(n)}(x) = E_n\psi_1^{(n)}(x). \tag{S7}$$

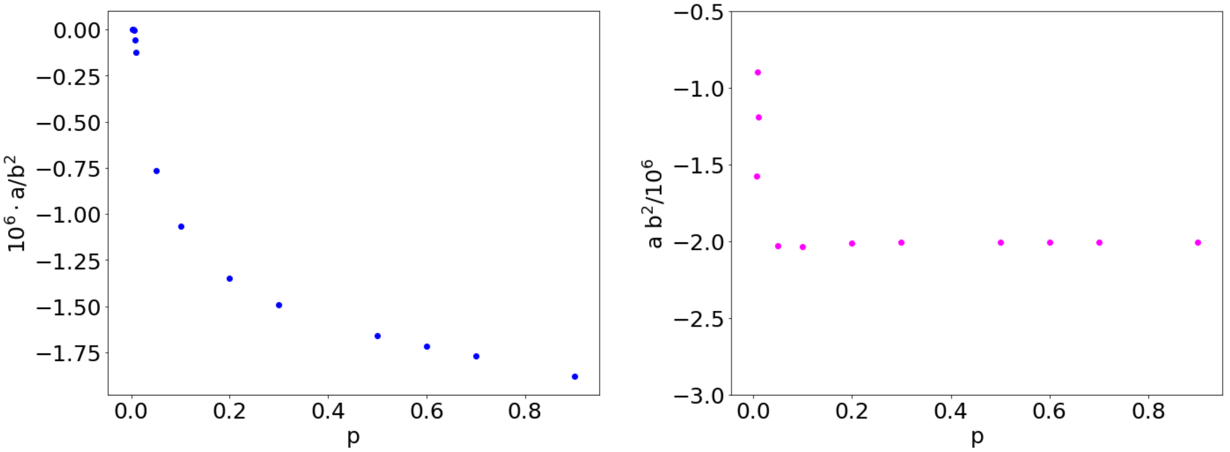

Figure S1. Left: a/b^2 as a function of p. Right: ab^2, as a function of $p \geq 6\times10^{-3}$.

Since, by construction, E_n is below the spectrum of p^2+V_{n+1}, the function f_n that satisfies (S5) yields a normalizable $\psi_1^{(n)}$ with no nodes, which is thus the ground state of p^2+V_n. The iteration proceeds until reaching $V_1(x)$, for which the lowest energy level is E_1, with eigenfunction $\psi_1^{(1)}$.

We are now ready to show that all the E_n's are also energy levels of V_1, and construct the corresponding eigenfunctions. The fundamental result is that, considering the relations (S6) and the commutator $[p, g(x)] = -\mathrm{i}g'(x)$,

$$(p^2+V_n)(p-\mathrm{i}f_n) = (p-\mathrm{i}f_n)(p^2+V_{n+1}). \tag{S8}$$

Due this relation, it is possible to verify that the normalizable wavefunctions

$$\psi_n^{(1)}(x) = \left[\prod_{j=1}^{n-1}(p-\mathrm{i}f_j(x))\right]\psi_1^{(n)}(x) \propto \left[\prod_{j=1}^{n-1}(\partial_x+f_j(x))\right]\exp\left(\int_0^x f_j(y)\mathrm{d}y\right) \tag{S9}$$

with $n>1$ and $\psi_1^{(n)}$ defined as in (S7), satisfy

$$(p^2+V_1(x))\psi_n^{(1)}(x) = E_n\psi_n^{(1)}(x). \tag{S10}$$

Additional properties of reconstructed potential

We add here a few comments on the structure of the Schrodinger potential associated with the Laplacian spectrum. If we expand the potential

$$V_{\text{fit}}(x,p) = a(p)\,\mathrm{sech}^2\left(\frac{x}{b(p)}\right) - 2 - a(p)\,, \tag{S11}$$

for $x \ll b$, we obtain

$$V_{\text{fit}}(x,p) = -2 - \frac{a}{b^2}x^2. \tag{S12}$$

The ration a/b^2 is therefore the concavity of the quadratic approximation (parabola) of the potential in the origin. Figure S1 (left) displays the behavior of this quantity vs p. Notice that it tends to vanish for $p \to 0$, as expected from Eq. (9) of the main text if $2\beta-\alpha>0$. Our fit yields $2\beta-\alpha \simeq 1$.

In Fig. S1 (right) we plot the quantity ab^2 vs p. This quantity is proportional to $\int_0^\infty xV(x)dx$ (in general, $\int_0^\infty x^nV(x)dx \propto ab^{n+1}$). Interestingly, it is almost constant for large values of p (far from the phase trasition). For smaller p it becomes very unstable (points $p<6\times10^{-3}$ not shown in the figure) and does not enable one to draw any solid conclusion.

Finally, we also observe that the functional form (S11) of V could be valid also at $p<p_c$. However, the presence of oscillations and the roughness of the median potential make the fit of a and b very difficult $p<p_c$.

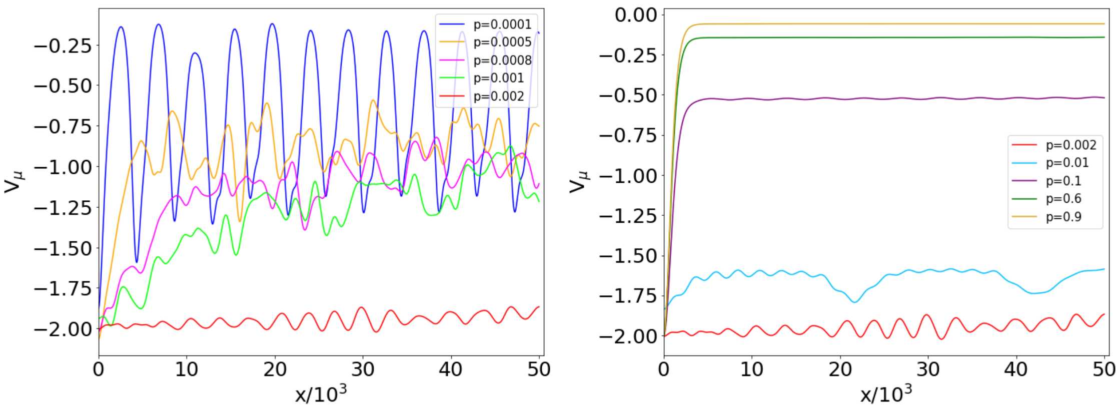

Figure S2. Pointwise mean $V_\mu(x)$ of the reconstructed potentials $V_i(x)$ $(i = 1, \ldots, M)$, for $M = 100$ ER networks, $N = 500$ nodes and connection probability p. The critical value is $p_c = 1/(N-1) \simeq 2 \times 10^{-3}$. Left panel: $p < p_c$, with p ranging from 10^{-4} (top) to 2×10^{-3} (bottom). Right panel: $p > p_c$, with p ranging from 2×10^{-3} (bottom) to 0.9 (top). The values of p are given in the insets, with the same color code as in Fig. 5 of the main text. For $p < p_c$, the mean potentials decrease as p approaches the critical value from below (left). For $p \gg p_c$, $V_\mu(x)$ rapidly increases and reaches a saturation value (right). Compare with Fig. 5 of the main text.

Comparing pointwise median and mean reconstructed potentials

Figure S2 shows the profile of the pointwise mean potential V_μ, evaluated at the same connection probabilities p reported in Fig. 5 of the main text for the pointwise median potential V_m. Although the overall trend of V_μ upon increasing p is reminiscent of the one observed for V_m, crucial differences between the two functions can be pointed out. First, by inspection of Fig.S2, it is evident that the profile of V_μ is always smooth, even when the median potential at the same probabilities exhibits a rugged shape. This observation is confirmed by the evaluation of the Higuchi Fractal Dimension (HFD), as defined in Section 3.3 of the main text. Actually, the HFD of the mean potential does not show any significant increase from 1, even in proximity of the percolation phase transition. Moreover, as shown in the left panel of Fig. S3 in the $p = 9 \times 10^{-4}$ case, the profiles of V_m and V_μ at a given connection probability p can also display macroscopic differences.

The aforementioned discrepancies between V_m and V_μ are signatures of the fact that median and mean generally encode non redundant information on the distribution of reconstructed potential values at fixed x. Actually, the occurrence of a very skew distribution for some x, as the one illustrated in the right panel of Fig. S3, leads a substantial separation between the values of $V_m(x)$ and $V_\mu(x)$, that remains relevant even for a very large number M of ER network realizations.

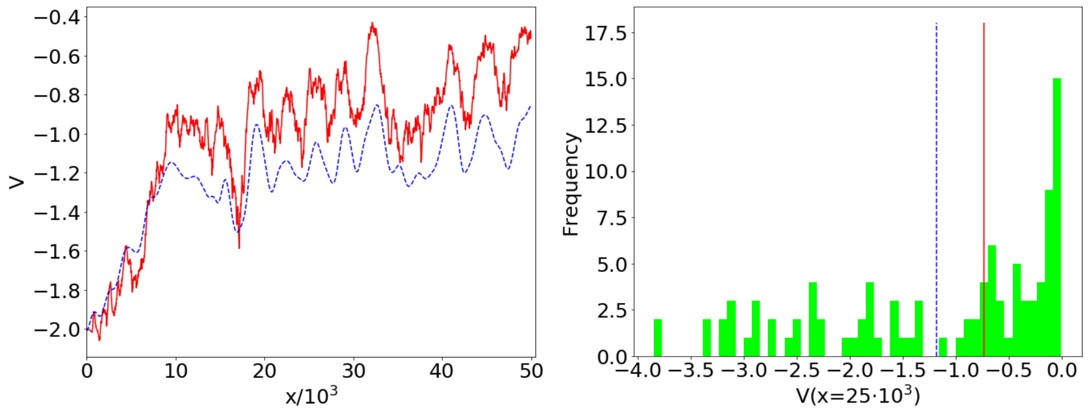

Figure S3. Left panel: pointwise median $V_m(x)$ (solid red line) and mean $V_\mu(x)$ (dashed blue line) potentials for $M = 100$ ER network realizations with $N = 500$ nodes, at fixed connection probability $p = 9 \times 10^{-4}$. Right panel: distribution of the reconstructed potential values at a fixed point $V_i(x = 25 \times 10^3)$ $(i = 1, \dots, M)$, with median (solid red line) and mean (dashed blue line) values highlighted.

Numerical procedure

In order to obtain M realizations of the ER networks at critical connection probability for all the considered values of N, it was necessary to split the computation on different servers, in order to reduce the computational time. This task was made possible by implementing parallel computing on the infrastructures provided by the Bari ReCaS Datacenter. For example, $M = 20000$ realizations of the ER networks with $N = 500$ nodes were analyzed by employing 400 different cores, each dedicated to the computation of reconstructed potentials related to 50 networks. On each core, the task was accomplished in about 24 hours: the same operations, run in series on a single device, would have required a 400 times longer computational time. All the reconstructed potentials associated to the 20000 random network realizations provided about 24 Gigabyte (Gb) data, that were subsequently transferred to a single server, to compute the median potential and its fractal dimension, yielding the results shown in Fig. 11 of the main text. The value of 24 Gb represents a limiting size for the amount of data that can be stored and processed simultaneously thru the 16 Gb RAM memory available on the single device. Therefore, it was not possible to extend the range of considered values of M and N, and make solid statements on the fractal dimension in the limits $M, N \to \infty$. The data show nonetheless that the fractality of the median potential detects the percolation phase transition.
